# Energy availability modulates regional blood flow via estrogen-independent pathways in regularly menstruating young women

**DOI:** 10.1007/s00421-024-05497-0

**Published:** 2024-05-29

**Authors:** Mark J. Hutson, Emma O’Donnell, Kyle McConnell, Aiden J. Chauntry, Richard C. Blagrove

**Affiliations:** 1 Faculty of Life and Health Sciences, School of Sport, Ulster University, Coleraine, BT52 1SA UK; 2 School of Sport, Exercise and Health Sciences, Loughborough University, Loughborough, LE11 3TU UK; 3 Department of Exercise and Sport Science, University of North Carolina at Chapel Hill, Chapel Hill, NC USA

**Keywords:** Energy restriction, Female health, Cardiovascular, Relative energy deficiency in sport

## Abstract

**Purpose:**

This study aimed to investigate the impact of short-term low energy availability (LEA) on vascular function in young, regularly menstruating women.

**Methods:**

Participants were 19 women, aged 22.9 ± 4.2 years, with body mass index 18–30 kg·m^2^. They were divided into two groups and completed two conditions in a crossover design: a 3-day control condition (CON) with an energy availability of 45 kcals·kgFFM^−1^·day^−1^ and a 3-day LEA condition of 15 kcals·kgFFM^−1^ day^–1^. Assessments were conducted during the early follicular phase of the menstrual cycle. Outcome measures included forearm blood flow (FBF), heart rate, blood pressure, arterial stiffness, resting energy expenditure (REE), metabolic blood markers and body composition.

**Results:**

Significant time-by-condition interactions were found for resting FBF (*p* = .004), REE (*p* = .042), triiodothyronine (*p* = .006), β-hydroxybutyrate (*p* = .002) and body mass (*p* < .001). Resting FBF was 1.43 ± 1.01 and 1.31 ± 0.61 (arbitrary units) at pre and post, respectively, in LEA and 1.52 ± 0.7 and 1.76 ± 0.57 at pre and post in CON. The LEA condition led to a decrease in triiodothyronine (pre: 1.54 ± 0.28, post: 1.29 ± 0.27 ng ml^−1^), REE (pre: 1588 ± 165, post: 1487 ± 160 kcals day^−1^) and body mass (pre: 61.4 ± 7.5, post: 59.6 ± 7.3 kg). Changes in resting FBF were significantly correlated with changes in REE in the LEA condition (*r* = 0.53; *p* = 0.02).

**Conclusion:**

Short-term LEA modifies regional blood flow and this might contribute to the observed decreased in REE. Findings emphasize the need for careful management of energy availability in populations at risk of LEA.

**Supplementary Information:**

The online version contains supplementary material available at 10.1007/s00421-024-05497-0.

## Introduction

Energy availability is the amount of energy that remains once exercise energy expenditure is deducted from energy intake and is considered low when optimal function of all remaining bodily processes cannot be maintained (Loucks et al. 2011). It is estimated that 45% of female endurance athletes and 23% of recreationally active females exhibit symptoms associated with low energy availability (LEA) (Logue et al. 2019; Melin et al. 2015). LEA lasting several months can cause menstrual disturbances such as amenorrhea (the cessation of menses for ≥ 90 days) via suppression of the hypothalamic–pituitary–ovarian axis, which is characterized by chronic estrogen deficiency (Areta et al. 2021; Williams et al. 2001, 2015). Exercising women with amenorrhea exhibit reduced resting energy expenditure (REE) commensurate with downregulated physiological function and energy conservation in response to LEA (Koehler et al. 2016). An association between LEA and impaired cardiovascular function exists in this population (O’Donnell et al. 2009, 2011, 2014).

Exercising women with amenorrhea and chronic estrogen deficiency have reduced resting systolic blood pressure, pulse pressure, heart rate, and endothelial-dependent and independent dilatory capacity at the brachial artery (O’Donnell et al. 2014). Paradoxically, vascular resistance is elevated despite lower resting blood pressure and resting and peak post-ischemic regional blood flow measured at the calf are reduced (O’Donnell et al. 2007). Endothelial function is regulated by estrogen receptor signaling (Pinna et al. 2008; Rubanyi et al. 1997), such that estrogen deficiency likely contributes to the vascular effects observed in exercising women with amenorrhea (Miller and Duckles 2008; O’Donnell et al. 2007). These findings hold importance given endothelial dysfunction is involved in cardiovascular disease progression (Forstermann and Munzel 2006).

LEA ≤ 15 fat-free mass^−1^ day^−1^ (kcals kgFFM^−1^ day^−1^) can cause acute endocrine perturbations such as suppressed triiodothyronine (T3) and leptin within just 3 days (Papageorgiou et al. 2018). In other contexts, these perturbations have also been associated with impaired endothelial and vascular function in vitro (Mizuma et al. 2001) and in human participants (Morioka et al. 2014). Estrogen concentrations remain stable during short-term LEA lasting up to 5 days in the low hormone phase of the menstrual cycle (Areta et al. 2021). This offers a window to investigate estrogen-independent effects of LEA on vascular function, which cannot be elucidated by comparing amenorrheic to eumenorrheic women given the coexistence of estrogen deficiency.

A more holistic understanding of the etiology and temporality of vascular effects of LEA is important for exercise performance, recovery, and health. Consequently, this study aimed to examine the effects of short-term LEA in regularly menstruating young women on resting and peak-ischemic blood flow at the forearm and other secondary outcomes associated with LEA or vascular function, including arterial stiffness, blood pressure, heart rate, REE, T3, body mass and composition. It was hypothesized that LEA would reduce resting and peak forearm blood flow compared to a more optimal level of energy availability, while estrogen concentration remained stable.

## Methods

### Participants and ethics

Participants were recruited using social media and word-of-mouth and met the following eligibility criteria: female, aged 18–40 years, body mass index 18–30 kg m^−2^, previous three menstrual cycles lasted 21–35 days, non-smoker, not used hormonal contraception or hormone replacement therapy in the previous 3 months, not vegan, never diagnosed with an eating disorder or medical condition known to impact menstrual function, and not currently dieting. Participants were asked if they regularly performed more than three vigorous or five moderate exercise sessions per week and were excluded if they responded “yes”, to minimize de-training effects during the trials. Twenty-one eligible young women provided informed consent. The study was registered at www.clinicaltrials.gov (NCT04790019) and received ethical approval from Loughborough University Ethics Review Sub-Committee.

### Experimental design

Participants completed a preliminary testing phase followed by two experimental trials which were completed during the early follicular phase of different menstrual cycles in a randomized and counterbalanced order. Trials were conducted during consecutive menstrual cycles or with one or two cycles between them. Pseudo-random online software was used to perform block randomization (Sealed Envelope Ltd.).

Preliminary testing included one laboratory visit to confirm eligibility, familiarization with REE methods, and completion of the Low Energy Availability in Females Questionnaire (LEAF-Q) (Melin et al. 2014). For the following 3 days, energy intake was measured using a weighed food diary, analyzed using nutritional software (Nutritics v5.64), and physical activity was monitored using a triaxial accelerometer (ActiGraph wGT3X-BT) worn on the non-dominant hip. Accelerations were collected at a sample rate of 90 Hz and analyzed (ActiLife v6.13.4) for average daily activity energy expenditure (DAEE) and time spent in moderate-to-vigorous physical activity (MVPA) using validated cut-points (Freedson et al. 1998).

### Experimental trials

Each trial started within 4 days following the self-reported onset of menses and involved 3 consecutive days at a set energy availability, either 45 (CON) or 15 kcals kgFFM^−1^ day^−1^ (LEA). Participants were instructed to avoid planned and structured exercise so that energy intake provided by the intervention diets was equal to energy availability. An accelerometer was worn during each trial to check for differences in MVPA which could indicate interference with energy availability manipulations. Omnivorous and vegetarian diets providing 45 kcals kgFFM^−1^ day^−1^ composed of 50% carbohydrate, 20% protein, and 30% fat were created for a reference individual (see supplementary file 1). The quantity of each ingredient was scaled to each participants pre-intervention FFM to produce CON diet and then divided by three to produce LEA diet. Omnivores and vegetarians were provided the diet that reflected their habitual practices. Ingredients were weighed to within 1 g (Mettler Toledo PL601-S Electronic Scale). Breakfast consumption was supervised at the laboratory and other meals were packaged. A daily multivitamin and mineral supplement (Vitawell A-Z Multivitamins & Minerals) was taken with breakfast during LEA and adherence to diets was confirmed verbally. Participants were instructed to eat all and only what was provided to them by the research team. Black coffee, black tea, and green tea were permitted to improve adherence (but not from midday prior to test visits) and fluid intake was recorded.

Tests were performed between 07:00 and 09:00 in a fasted state on the first morning of each trial and repeated at the same time on the morning after the last day of each trial. Ambient laboratory temperature, humidity, and pressure were 21.6 ± 0.4 °C, 35.6 ± 3.7%, and 1016 ± 4 mmHg, and there were no significant differences between time points (*p* > 0.309). Body mass and composition were measured using bioelectrical impedance scales (Seca MBCA 515). Further data were collected using indirect calorimetry, brachial sphygmomanometry, pulse wave analysis, venous occlusion plethysmography, then blood sampling. Participants were instructed to avoid strenuous exercise, alcohol, caffeine from midday prior to test visits, and were asked to record and replicate their diet for the 24 h prior to pre-tests in each trial. Participants were provided the same frozen pizza (https://web.archive.org/web/20220116215841/tesco.com/groceries/en-GB/products/291196322) to eat between 19:00 and 20:00 the evening prior to all pre and post-tests. Frozen pizza quantities provided the evening prior to post-tests (704 ± 45 kcals in CON and 233 ± 13 kcals in LEA) were calculated as part of the intervention diet using the process described previously. For the evening prior to pre-tests, the quantity provided matched that of the final day of the control condition. Participants drank 500 ml of water upon waking on the morning of each test visit and a urine sample of their first void was analyzed for urine specific gravity (USG) using a handheld refractometer. USG and change in plasma volume were considered indicators of hydration status, primarily to aid interpretation of changes in body composition. Whole blood was sampled using the methods described below and used to estimate percentage pre to post-test plasma volume change using the cyanmethemoglobin method (Dill and Costill 1974). Participants completed the Pittsburgh Sleep Quality Index (PSQI) during pre-tests to produce a cumulative sleep quality score for the previous month.

### Indirect calorimetry

REE was assessed via indirect calorimetry in a dark, quiet, temperature-controlled laboratory. Participants lay at rest for 15-min, prior to a mouthpiece and nose clip being fitted, followed by a further 5-min of familiarization with the breathing apparatus. Expired and ambient air were then collected simultaneously in separate Douglas bags for 5-min and used to determine $\dot{V}$O_2_ and $\dot{V}$CO_2_. Percentage O_2_ and CO_2_ in expired and ambient samples were analyzed using a gas analysis system (Servomex 1440), calibrated in triplicate on the same day. Total expired volume was measured using a dry gas meter (Harvard Ltd) and standardized to air temperature, ambient pressure, and vapor pressure. The Weir equation was used to calculate REE in kcals day^−1^. Respiratory exchange ratio (RER) was calculated as $\dot{V}$CO_2_/$\dot{V}$O_2_ for the duration of the gas collection period.

### Brachial sphygmomanometry

Participants remained at rest while vascular measures were taken. Resting heart rate plus brachial systolic and diastolic blood pressures were measured in triplicate using a sphygmomanometer (OMRON M7 Intelli IT) and used to calculate mean arterial pressure (MAP). First readings were discarded and an average of the final two was calculated.

### Pulse wave analysis

To assess arterial stiffness, pulse wave analysis was performed at the radial artery using a tonometric device and software (SphygmoCor©, AtCor Medical Pty Ltd.). Measures of interest included augmentation index and augmentation index normalized to a HR of 75 bpm (AIx75). Recordings were taken in duplicate, and triplicate if AIx75 differed by > 4%; the average of the closest two was used (Stoner et al. 2012). Operator index was ≥ 80 for all recordings.

### Venous occlusion plethysmography

Resting and peak FBF was measured using venous occlusion plethysmography techniques, see (Wilkinson and Webb 2001) for a detail review of the method. An appropriately sized 4-wire mercury-in-silastic strain gauge, fitted around the widest part of the forearm, was connected to a plethysmograph (Hokanson EC6, Washington, USA) that had been calibrated using a 2-point volume system: 0 and 1%. A venous occlusion cuff (Hokanson SC10D) attached to a rapid cuff inflation system (Hokanson E20) was placed around the upper arm proximal to the elbow joint and the forearm was positioned slightly above heart level. A wrist cuff was inflated to > 200 mmHg throughout FBF measurements to exclude hand blood flow. The forearm cuff was inflated to 50 mmHg for 7-s (to interrupt venous emptying) and then deflated for 7-s to allow venous emptying. Arterial inflow is proportional to the linear increase in forearm volume during cuff inflation (Greenfield et al. 1963). Five successive cuff inflations and deflations were performed. Percentage increase in forearm volume per minute (%FV min^−1^) over the first three complete and consecutive cardiac cycles following each cuff inflation was analyzed using commercial software (ADInstruments LabChart v8.1.13) and the average was calculated for resting FBF. The same process was then repeated following 5-min of cuff inflation to 200 mmHg, causing forearm ischemia. The first post-ischemic measurement was taken swiftly as possible following deflation from 200 mmHg (i.e., during reactive hyperemia) and %FV min^−1^ during the first complete cardiac cycle measured was analyzed for peak FBF (Junejo et al. 2019). Between-day bias ± 95% limits of agreement for resting and peak FBF was previously estimated in our laboratory as – 0.2 ± 0.5 and -1.2 ± 3.4%FV min^−1^, respectively, in a sample of 12 young healthy males and females (unpublished data)—comparable to previous research (Thijssen et al. 2005). Area under the curve (AUC) during the first complete cardiac cycle was calculated using the trapezoidal method for all five post-ischemic recordings and summed to indicate FBF recovery rate.

### Biochemical sampling and analysis

Blood was drawn from an antecubital forearm vein between 09:00–10:00. Samples were collected in K2EDTA and serum separation tubes (BD Vacutainer®, Franklin Lakes, USA) and stored on ice (plasma) or at room temperature (serum) for 30-min before centrifugation at 2,058 G for 15-min at 4 °C. Samples were stored at -80 °C for later analysis. Total T3 and 17β-oestradiol were measured in serum and β-hydroxybutyrate (β-OHB) in plasma. T3 was measured using an automated electro-chemiluminescence immunoassay analyzer (Roche Diagnostics Cobas e411). Inter-assay coefficient of variation (CV) was 3.4%. 17β-oestradiol (intra-assay CVs: 8.3 and 7.8%, inter-assay CV: 8.0%) was measured using a manual enzyme-linked immunosorbent assay as per manufacturers’ instructions (IBL International GmbH). β-OHB (inter-assay CV: 10.2%) was analyzed using an enzymatic spectrophotometric assay (Randox) as per manufacturers’ instructions. All times points for individual subjects were analyzed on the same plate.

### Statistical analysis

Data are presented as mean ± standard deviation (SD) unless stated otherwise. Residuals were checked for normality using the Shapiro–Wilk test, cases ≥ 3 SD outside the mean were considered outliers and removed prior to analysis. Non-normal data were transformed prior to analysis using the natural logarithm such that the assumption of normality was not violated. Data are presented as median ± interquartile range for all variables that were transformed prior to analysis. Paired *t*-test was used to check for differences in measured extraneous variables between CON and LEA. Two-way repeated measures ANOVA was used to investigate time by condition interaction effect for all outcome measures. Partial eta-squared was reported to indicate interaction effect size (*η*_*p*_^2^). Pearson correlation was used to identify relationships between variables and all data included in correlations were normally distributed. Data were analyzed using SPSS version 27 (IBM, Chicago, USA) and alpha was set as < 0.05. Cases with missing data were excluded listwise.

## Results

Participant characteristics are presented in Table 1. Two participants dropped out after completing one condition: one started using oral contraception and the other re-located. Ambient laboratory temperature, humidity, and pressure were 21.6 ± 0.4°C, 35.6 ± 3.7%, and 1016 ± 4 mmHg, and there were no significant differences between time points (*p* > 0.309). There was a significant correlation between daily EI and activity exercise expenditure at preliminary testing phase (*r* = 0.75, *p* < 0.001).

**Table 1:** Participant characteristics at preliminary testing phase

Age (years)	Height (m)	Body mass (kg)	LEAF-Q score	Average daily EI (kcals)	Average DAEE (kcals)
22.9 ± 4.2	1.64 ± 0.06	61.2 ± 8.0	4.5 ± 3.1	1866 ± 504	452 ± 233

There were no significant differences in CON vs. LEA for PSQI score (both 4.6 ± 1.0, *p* > 0.999), MVPA (52 ± 33 vs. 57 ± 30 min, *p* = 0.438), DAEE (417 ± 213 vs. 439 ± 235 kcals, *p* = 0.570), daily water intake (2.33 ± 1.10 vs. 2.49 ± 0.92 L, *p* = 0.230), or pre-post change in plasma volume (– 2.9 ± 4.9 vs. – 0.3 ± 5.0%, *p* = 0.065).

**Fig. 1:**
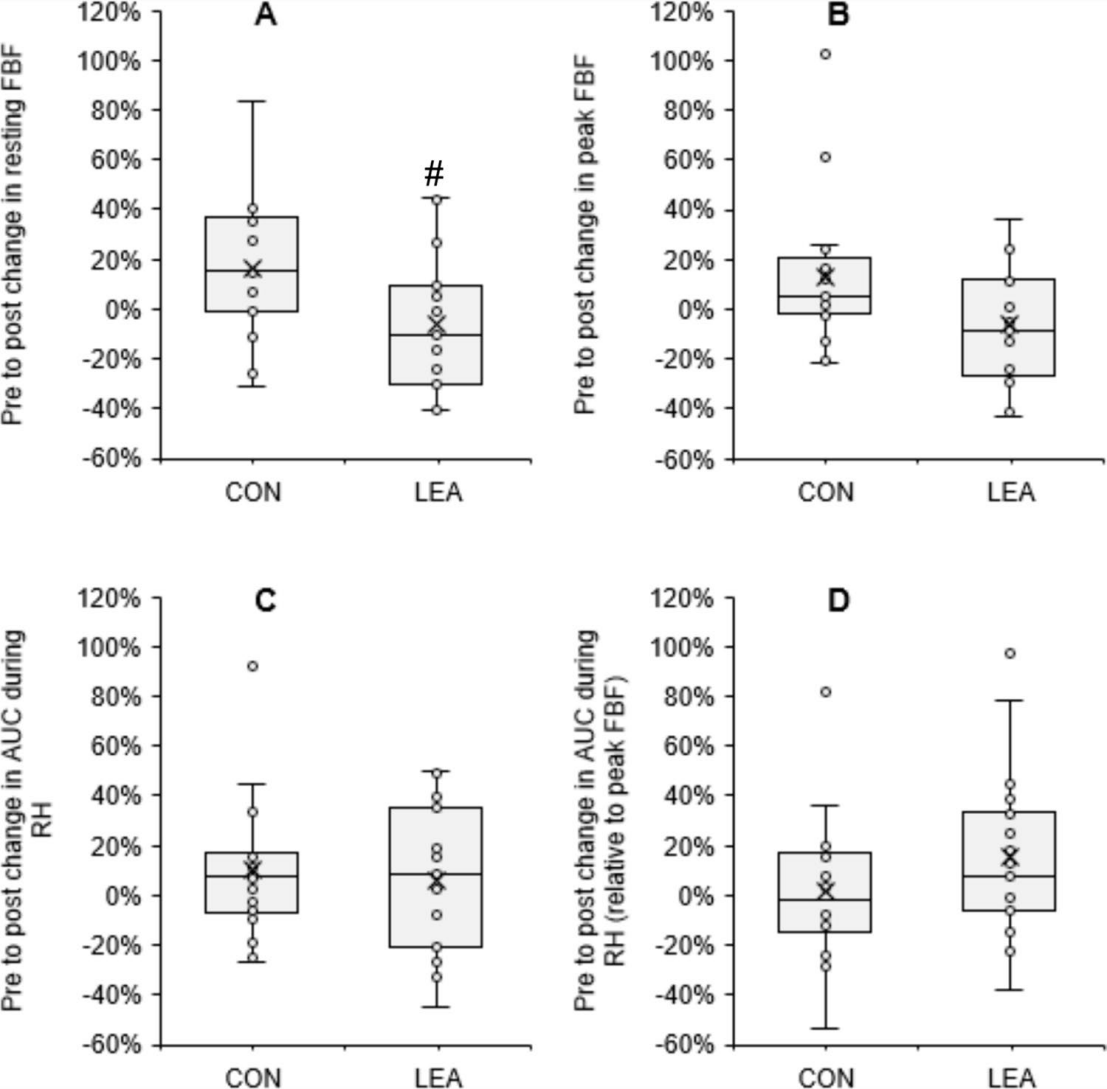
Box and whisker plots showing percentage change from pre to post (%) in control (CON) and low energy availability (LEA) conditions for **A** resting forearm blood flow (FBF), **B** peak FBF, **C** AUC during reactive hyperemia (RH), and **D** AUC during reactive hyperemia relative to peak FBF. Individual data points are shown by circles and crosses shown the mean change. Significant difference compared to CON is indicated (#)

**Fig. 2:**
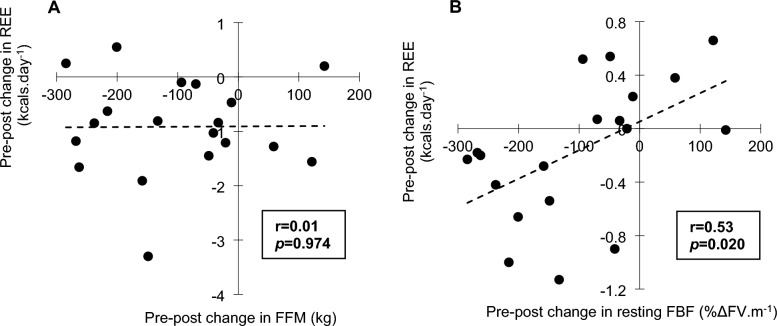
Scatter plots and Pearson’s correlations for pre-post change in the low energy availability condition between **A** resting energy expenditure (REE) and fat-free mass (FFM), and **B** REE and resting forearm blood flow (FBF). Correlation coefficients (r) and associated *p*-values are reported

Data and statistical interaction effects regarding body composition, urine specific gravity, metabolic and hormonal markers, and cardiovascular outcomes are presented in Table 2. There were significant time by condition interactions for body mass, FFM, fat-mass, T3, β-OHB, and REE. There was also a significant time by condition interaction for resting FBF (shown in Fig. 1), but not for 17β-oestradiol, peak FBF, FBF AUC during reactive hyperemia, or any other cardiovascular outcome. Pre-post change in the LEA condition for resting FBF was significantly correlated with that of REE (*r* = 0.52, *p* = 0.020, see Fig. 2B), but not with that of T3 (*r* = 0.01, *p* = 0.968). There was also no significant correlation between REE and FFM (*r* < 0.01, *p* = 0.974, see Fig. 2A) or FFM and resting FBF (*r* = – 0.01, *p* = 0.971).

**Table 2:** Body composition, urine specific gravity, metabolic and hormonal, and cardiovascular outcomes at each time point, and corresponding time by condition interaction effects

Outcome	Condition	Pre	Post	*η*_p_^2^	*p*-value
Body composition					
Body mass (kg)	CON	60.7 ± 6.8	60.4 ± 7.3	**0.573**	** < 0.001**
	LEA	**61.4 ± 7.5**	**59.6 ± 7.3**^**c**^		
Fat-free mass (kg)	CON	44.7 ± 3.6	44.5 ± 3.6	**0.32**	**0.009**
	LEA	**45.0 ± 3.7**	**44.1 ± 3.6**^**c**^		
Fat mass (kg)	CON	16.0 ± 4.9	15.9 ± 5.3	**0.28**	**0.017**
	LEA	**16.3 ± 5.2**	**15.5 ± 5.4**^**c**^		
Hydration					
^Ln^ Urine specific gravity	CON	1.024 ± 0.014	1.022 ± 0.013	0.01	0.683
	LEA	1.022 ± 0.011	1.025 ± 0.013		
Metabolic and hormonal					
Resting energy expenditure (kcals.day^−1^)	CON	1503 ± 121	1489 ± 150	**0.21**	**0.042**
	LEA	**1588 ± 165**	**1487 ± 160**^**b**^		
Resting respiratory exchange ratio	CON	0.84 ± 0.07	0.83 ± 0.07	0.13	0.113
	LEA	**0.80 ± 0.04**	**0.75 ± 0.04**^**b**^		
^^^^ Total triiodothyronine (ng.ml^−1^)	CON	1.50 ± 0.26	1.49 ± 0.25	**0.38**	**0.006**
	LEA	**1.54 ± 0.28**	**1.29 ± 0.27**^**b**^		
^^^^^ β-hydroxybutyrate (mmol.l^−1^)	CON	0.21 ± 0.11	0.22 ± 0.10	**0.47**	**0.002**
	LEA	**0.19 ± 0.10**	**0.54 ± 0.35**^**c**^		
^^^, Ln^ 17β-oestradiol (pg.ml^−1^)	CON	79.0 ± 39.0	70.9 ± 33.9	0.01	0.725
	LEA	83.0 ± 39.0	76.0 ± 60.2		
Cardiovascular					
Resting heart rate (beats.min^−1^)	CON	56 ± 10	56 ± 8	0.14	0.110
	LEA	**58 ± 10**	**56 ± 10**^**b**^		
Systolic blood pressure (mmHg)	CON	106 ± 7	103 ± 5	< 0.01	0.846
	LEA	106 ± 6	104 ± 6		
^#^ Diastolic blood pressure (mmHg)	CON	**66 ± 6**	**63 ± 5**^**a**^	0.02	0.565
	LEA	66 ± 6	65 ± 4		
^#^ Mean arterial pressure (mmHg)	CON	**79 ± 6**	**77 ± 5**^**a**^	0.02	0.593
	LEA	80 ± 5	78 ± 3		
^Ln^ Pulse pressure (mmHg)	CON	25 ± 6	26 ± 4	0.02	0.562
	LEA	26 ± 5	25 ± 6		
Augmentation index at 75 bpm (%)	CON	1.1 ± 10.4	-0.9 ± 8.0	< 0.01	0.952
	LEA	1.0 ± 9.3	-1.1 ± 9.6		
^Ln^ Resting blood flow (AU, %ΔFV.min^−1^)	CON	1.52 ± 0.78	1.76 ± 0.57	**0.38**	**0.004**
	LEA	1.43 ± 1.01	1.31 ± 0.61		
^^^ Peak blood flow (AU, %ΔFV.min^−1^)	CON	20.8 ± 6.4	22.4 ± 5.3	0.17	0.085
	LEA	20.0 ± 6.3	18.4 ± 5.6		
^^^ Reactive hyperemia AUC (AU)	CON	34.9 ± 9.4	37.5 ± 10.7	< 0.01	0.836
	LEA	34.5 ± 10.7	36.3 ± 9.5		
^^, Ln^ Reactive hyperemia AUC/Peak blood flow (AU)	CON	1.60 ± 1.43	1.64 ± 0.97	0.10	0.193
	LEA	**1.77 ± 0.68**	**2.00 ± 1.85**^**a**^		

Partial eta-squared has been reported to indicate effect size (*η*_p_^2^) and significant within condition (post vs. pre) post-hoc comparisons are shown in bold: ^a^*p* < 0.05, ^b^*p* < 0.01, ^c^*p* < 0.001

^#^1 case with outlier removed prior to analysis

^^^1 case with missing data

^^^^2 cases with missing data

^^^^^3 cases with missing data

^Ln^Variable log transformed prior to analysis and data are presented as median ± IQR

## Discussion

This is the first prospective trial on the effect of severe LEA or dietary energy restriction on vascular function in young healthy women. For the first time, we demonstrate that in eumenorrheic non-exercising women assessed during the low-hormone phase of the menstrual cycle, resting FBF is decreased with just 3-days LEA. We also identify an association between resting FBF and REE, which may offer mechanistic insights into metabolic allostasis in response to LEA (Areta et al. 2021; Koehler et al. 2016).

### Resting blood flow

We show that energy availability significantly decreases resting FBF within just 3 days. This may have important health implications for young women. Impaired FBF has been associated with hypertension and increased risk of adverse cardiovascular events (Higashi et al. 2002; Pedrinelli et al. 1995; Perticone et al. 2001). Long-term LEA can cause chronic estrogen deficiency, with amenorrhea often indicating long-term LEA in active women (Heikura et al. 2018; Williams et al. 2001). Previous research has found that, compared to eumenorrheic counterparts, exercising women with amenorrhea have lower resting calf blood flow (O’Donnell et al. 2014), impaired endothelial dependent and independent dilatory function (Rickenlund et al. 2005; Yoshida et al. 2006), and augmented calf vascular resistance (O’Donnell et al. 2009). Estrogen deficiency, in association with reduced NOS activation and nitric oxide release, is postulated to play a key role (O’Donnell et al. 2011). However, given our data were collected during the early follicular phase of the menstrual cycle (i.e., the low hormone phase) we suggest that vascular impairments in young premenopausal women may be initiated by LEA, prior to observable reductions in 17β-oestradiol. Future studies may wish to examine whether nitric oxide signaling remains a key factor independent of changes in 17β-oestradiol. Nevertheless, our observed resting FBF was only ~ 26% lower following three days of LEA at 15 versus 45 kcals kgFFM^−1^ day^−1^, compared to 48% lower resting calf blood flow on average in amenorrheic athletes versus eumenorrheic counterparts (O’Donnell et al. 2014). Differences may be somewhat confounded by measurement site; however, previous research has found little difference in baseline resting blood flows between the two sites (Nishiyasu et al. 1992). It is likely that LEA combined with chronic estrogen deficiency exerts additive adverse effects on regional blood flow in exercising women (O’Donnell et al. 2007), and that this at least contributes to the differences described, but further investigations are needed.

Resting FBF exhibited a significantly different change during LEA compared to CON; however, post-hoc comparisons in both conditions were not significant. Although measured using different methods, brachial blood flow has been shown to exhibit a natural increase of ~ 26% from early to late follicular phase of the menstrual cycle in young regularly menstruating women (Adkisson et al. 2010; Gavin et al. 2009), and this might explain the average increase observed during CON. In this context, the interaction effect is an interesting and significant finding given there was no effect on circulating estrogen. The prevention of an increase in resting FBF during LEA was likely linked with other hormonal and metabolic factors associated with short-term energy restriction and vascular function, such as: leptin (Vecchione et al. 2002), IGF-1 (Izhar et al. 2000), SIRT-1 (Gonçalinho et al. 2023), or T3 (Napoli et al. 2007). In agreement with previous research, we did not observe a relationship between resting FBF and T3 (O’Donnell et al. 2019); however, T3 infusion has been shown to enhance brachial artery endothelial regulation of vascular tone and resultant blood flow (Napoli et al. 2007). The current study was not designed specifically to look at a relationship between T3 and FBF and a mechanistic role of T3 should not be dismissed based on our findings.

### Peak blood flow

In addition to the interaction observed in resting FBF, a similar pattern of change was noted for peak FBF, but the effect of LEA on peak FBF was not statistically significant and there was large interindividual variability. It could be suggested that the study may have been underpowered to detect an effect; however, the conclusion that short-term LEA does not impair peak FBF is consistent with previous research showing that flow mediated dilation (peak dilatory response to augmented flow, indicative of endothelial function) was unaffected by two days of severe energy restriction in healthy men (fed 600 kcals day^−1^) and women (fed 500 kcals day^−1^) (Headland et al. 2019). Furthermore, evidence from exercising women with LEA-associated menstrual disturbances such as anovulatory menstrual cycles or amenorrhea lasting ≤ 100 days, suggests that peak calf blood flow may be preserved compared to eumenorrheic counterparts (O’Donnell et al. 2007). It seems that peak blood flow is more robust to the effects of LEA than resting blood flow. This hypothesis aligns with the life history perspective on LEA, as it would have been an evolutionary advantage to be able to maintain peak blood flow and support intense physical activity (i.e., hunting) during times of food scarcity (Shirley et al. 2022). Nevertheless, peak calf blood flow is significantly lower in exercising women with amenorrhea lasting > 100 days compared to eumenorrheic counterparts (O’Donnell et al. 2007). Estrogen deficiency elicits transcriptional and translational effects and can cause endothelial dysfunction, accelerated vascular smooth muscle proliferation, and vessel stiffening (Mendelsohn and Karas 1999). Prolonged exposure to estrogen deficiency may, over time, exert structural limitations to minimum vascular resistance and peak regional blood flow that cannot be overcome.

Our data suggest that short-term LEA does not modify vascular recovery from post-ischemic peak FBF; however, no other data exists in short or longer-term models of LEA by which to compare these findings. Disrupted cardiovascular recovery following different stimuli (such as exercise and psychological stress) has been associated with cardiovascular disease and sudden cardiac death (Panaite et al. 2015; Qiu et al. 2017; Steptoe and Marmot 2005), and future studies should explore cardiovascular recovery following longer bouts of LEA.

### Physiological function and body composition

T3, β-OHB, and glucose all exhibited changes comparable to those of previous research, which have investigated the effects of three to five days LEA in young females, indicating that participants were compliant and LEA was induced (Areta et al. 2021). This was accompanied by a loss of 1.8 ± 0.6 kg body mass, comparable to 1.8 kg and 1.6 kg lost following three- and five-days LEA, respectively, in similar samples (Papageorgiou et al. 2017, 2018). The current data do not indicate any major pre-post differences in hydration status; however, other plausible explanations for a rapid loss of body mass include reduced muscle glycogen and residual gut contents. These data support that participants were not energy deficient upon entry to the study, despite that reported energy intake at the preliminary testing phase seemed lower than total energy expenditure (estimated by summing REE, DAEE, and an additional 10% for dietary induced thermogenesis). However, this is based on group averages and the positive correlation between energy intake and DAEE suggests that those that expended more energy in exercise also had greater energy intakes, and vice versa. Furthermore, average underreporting when using food diaries has been estimated at 18% (Hill and Davies 2001). This equates to ~ 336 kcals in the current study and might also explain the disparity between energy intake and estimated total expenditure during preliminary testing.

Energy restriction has been found to result in physiological adaptations, such as decreased metabolic rate, that reduce daily energy expenditure and the rate of body mass loss (Silva et al. 2018), a phenomenon widely referred to as adaptive thermogenesis (Müller et al. 2015). To the best of our knowledge, we are the first to show a significant reduction in REE (– 100 ± 128 kcals.day^−1^) under tightly controlled laboratory conditions, during just three days of LEA. RER was > 0.7 and < 1 for all participants at every measurement point. RER reduced from pre to post in LEA and this was likely underpinned by reduced endogenous glycogen stores due to reduced dietary carbohydrate availability (Kojima et al. 2020). We found no correlation between change in REE and FFM during LEA and suggest that downregulated tissue function (not loss of mass) may be responsible. In support of this, previous research has shown that lower REE in exercise-associated amenorrhea is not due to reduced tissue mass (Koehler et al. 2016). These findings identify that metabolic allostasis can occur rapidly during LEA and underscore the importance of using measures other than changes in body mass to indicate energy status. Furthermore, the positive association between resting FBF and REE raises questions relating to the etiology of metabolic allostasis during LEA. Causality cannot be implied; however, future studies should explore whether endothelial and vascular function are involved in conserving energy during LEA by regulating downstream tissue function or core area kept at body temperature.

### Limitations

This study has several strengths, including a robust study design, a valid control condition, detailed measurement of extraneous variables, and the provision of freshly prepared food. There are also some limitations. The sample size is relatively small, but still larger than other studies investigating the effects of LEA utilizing similar methodologies (Areta et al. 2021). We were unable to estimate an effect size for a priori power calculation given the novelty of this study and analyses should therefore be considered exploratory. Our sample comprised young, regularly menstruating women that were studied exclusively during the early follicular phase and did not perform more than three vigorous or five moderate exercise sessions per week. This limits the generalizability of the findings to other menstrual cycle phases and populations, including athletes. We were not able to identify a specific mechanism for alterations in vascular function observed. Additionally, exercise was avoided during both trials to avoid the known endothelial nitric oxide stimulatory effects of exercise-induced increases in shear stress (O’Donnell et al. 2014). As such, vascular responses to LEA induced by exercise expenditure could not be determined in the current study. Although the data suggest otherwise, the reliance on self-adherence to dietary protocols may have compromised internal validity. Future research should consider a more diverse and larger sample size, a wider hormonal and metabolic assessment, concomitant exercise, and stricter methods for dietary adherence.

## Conclusion

In conclusion, the data suggest that 3-days of LEA may impact vascular function in young, regularly menstruating women. In addition to the recognized importance of estrogen to vascular health in women, our findings suggest that LEA may independently induce vascular impairments prior to perturbations in estrogen status. This may influence the health and performance of female athletes. Appropriate monitoring of energy availability in the female athlete may help prevent such vascular perturbations. Furthermore, healthcare practitioners should be aware that reduced regional blood flows may be associated with acute or chronic LEA. Further studies to explore the complex relationship between energy availability, hormonal regulation, and cardiovascular health in a broader demographic are warranted.

## Supplementary Information

Below is the link to the electronic supplementary material.

Supplementary file1 (DOCX 15 KB)

## Data Availability

The datasets generated during and/or analyzed during the current study are available from the corresponding author on reasonable request.

## Abbreviations

%FV min-1: Percentage increase in forearm volume per minute
AUC: Area under the curve
AIx75: Augmentation index normalized to a HR of 75 bpm
β-OHB: β-Hydroxybutyrate
CON: Control
DAEE: Daily activity energy expenditure
FBF: Forearm blood flow
kcals kgFFM^–1^ day^–1^: Kilocalories per kilogram of fat-free mass per day
LEA: Low energy availability
LEAF-Q: Low energy availability in females questionnaire
MAP: Mean arterial pressure
MVPA: Moderate-to-vigorous physical activity
PSQI: Pittsburgh sleep quality index
REE: Resting energy expenditure
T3: Triiodothyronine
USG: Urine specific gravity
