# Correction to: Energy availability modulates regional blood flow via estrogen‑independent pathways in regularly menstruating young women

**DOI:** 10.1007/s00421-024-05547-7

**Published:** 2024-08-10

**Authors:** Mark J. Hutson, Emma O’Donnell, Kyle McConnell, Aiden J. Chauntry, Richard C. Blagrove

**Affiliations:** 1 Faculty of Life and Health Sciences, School of Sport, Ulster University, Coleraine, BT52 1SA UK; 2 School of Sport, Exercise and Health Sciences, Loughborough University, Loughborough, LE11 3TU UK; 3 Department of Exercise and Sport Science, University of North Carolina at Chapel Hill, Chapel Hill, NC USA

**Correction to: European Journal of Applied Physiology** 10.1007/s00421-024-05497-0

In the original version of this article, reference citations in the text do not correspond to the correct sentences.

The original article has been corrected.

