# Supplementary material for: Energy availability modulates regional blood flow via estrogen-independent pathways in regularly menstruating young women
Source: Eur J Appl Physiol. 2024 May 29;124(10):3045–55. doi: 10.1007/s00421-024-05497-0 (PMC11466997; doi:10.1007/s00421-024-05497-0)
Supplement: Supplementary file 1 — Supplementary file1 (DOCX 15 KB) [file 421_2024_5497_MOESM1_ESM.docx]

**Supplementary file 2:** A breakdown of the ingredients and quantities used in each of the experimental diets.

**Experimental diets:**

Ingredients and quantities to provide 45 kcals·kg^-1^fat-free mass·day_-1_ to an individual with 45 kg fat-free mass.

Diet for omnivorous participants:

Day 1 (2,037 kcals, 254 g carbohydrate, 99 g protein, 68 g fat):

**Breakfast:** 60 g corn flakes with 258 ml full fat milk, 180 g Arla Skyr simply natural yogurt, 200 ml fresh orange juice.

**Lunch:** 150 g Baguette with 50 g cheddar cheese and 45 g tomato.

**Snack:** 190 g chopped apple and 30 g almonds.

**Dinner:** 100 g beef mince (~12 % fat), 4.2 g (1 tsp) olive oil, 50 g onion, 50 g red kidney beans, 150 g tinned chopped tomatoes, 10 g Schwartz chilli con carne spice blend and 150 g microwavable long grain rice.

Day 2 (2,030 kcals, 251 g carbohydrate, 99 g protein, 69 g fat):

**Breakfast:** 60 g corn flakes with 227 ml full fat milk, 120 g Arla Skyr simply natural yogurt, 350 ml fresh orange juice.

**Lunch:** 150 g Baguette with 100 g egg and 14 g butter.

**Snack:** 180 g chopped apple and 30 g almonds.

**Dinner:** 90 g chicken breast, 4.2 g (1 tsp) olive oil, 60 g broccoli, 80 g red pepper, 50 g soft cheese and 100 g fusilli pasta.

Day 3 (2,035 kcals, 252 g carbohydrate, 100 g protein, 68 g fat):

**Breakfast:** 50 g corn flakes with 227 ml full fat milk, 130 g Arla Skyr simply natural yogurt.

**Lunch:** 140 g Baguette with 100 g egg and 10 g butter.

**Snack:** 120 g chopped apple, 30 g almonds and 120 g Arla Skyr simply natural yogurt.

**Dinner:** 380 g stonebaked mixed vegetable pizza (Tesco).

Diet for vegetarian participants:

Day 1 (2,037 kcals, 253 g carbohydrate, 99 g protein, 68 g fat):

**Breakfast:** 60 g corn flakes with 258 ml full fat milk, 180 g Arla Skyr simply natural yogurt, 200 ml fresh orange juice.

**Lunch:** 150 g Baguette with 60 g cheddar cheese and 45 g tomato.

**Snack:** 180 g chopped apple and 40 g almonds.

**Dinner:** 100 g Quorn mince, 6.3 g olive oil, 50 g onion, 50 g red kidney beans, 150 g tinned chopped tomatoes, 10 g Schwartz chilli con carne spice blend and 130 g microwavable long grain rice.

Day 2 (2,038 kcals, 253 g carbohydrate, 99 g protein, 69 g fat):

**Breakfast:** 60 g corn flakes with 258 ml full fat milk, 200 g Arla Skyr simply natural yogurt, 300 ml fresh orange juice.

**Lunch:** 150 g Baguette with 100 g egg and 12 g butter.

**Snack:** 160 g chopped apple and 30 g almonds.

**Dinner:** 100 g Quorn chicken pieces, 4.2 g (1 tsp) olive oil, 60 g broccoli, 80 g red pepper, 50 g soft cheese and 100 g fusilli pasta.

Day 3 (2,035 kcals, 252 g carbohydrate, 100 g protein, 69 g fat):

**Breakfast:** 50 g corn flakes with 227 ml full fat milk, 130 g Arla Skyr simply natural yogurt.

**Lunch:** 140 g Baguette with 100 g egg and 10 g butter.

**Snack:** 120 g chopped apple, 30 g almonds and 120 g Arla Skyr simply natural yogurt.

**Dinner:** 380 g stonebaked mixed vegetable pizza (Tesco).

Lactose containing ingredients were substituted for lactose-free alternatives for two lactose-intolerant participants and total kilocalorie content and macronutrient composition were kept the same.
